# Medicinal Plant-Derived Exosome-like Nanoparticles: From Basic Research to Biomedical Applications

**DOI:** 10.3390/pharmaceutics18060750

**Published:** 2026-06-18

**Authors:** Huan Deng, Yi-Wen Zhang, Qian-Fu Zhao, Zhi-Jun Huang

**Affiliations:** School of Chemistry, Chemical Engineering and Life Sciences, Wuhan University of Technology, Wuhan 430070, China; denghuan@whut.edu.cn (H.D.); 374851@whut.edu.cn (Y.-W.Z.); zhaoqianfu2024@whut.edu.cn (Q.-F.Z.)

**Keywords:** plant-derived exosome-like nanoparticles, extracellular vesicle, ross-kingdom regulation, therapeutic applications

## Abstract

Plant-derived exosome-like nanoparticles (PELNs), a subset of extracellular vesicle (EV) secreted by plant cells, have emerged as revolutionary biomaterial with broad applications in biomedicine, agriculture, and nanotechnology. Structurally, PELNs feature a phospholipid bilayer homologous to plant cell membranes, encapsulating bioactive components such as proteins, nucleic acids, lipids, and secondary metabolites. The native structure of PELNs endows them with enhanced bioavailability, reduced immunogenicity, and improved barrier penetration for precise tissue delivery. Recent studies highlight the cross-kingdom therapeutic potential of PELNs in mammals, including antitumor, anti-inflammatory, tissue repair, immunomodulation and so on. This review comprehensively summarized recent advancements in PELN research, including innovative isolation techniques, molecular characterization, their roles in drug delivery and disease therapy. We also discussed challenges in standardization, scalability, and regulatory frameworks which could provide future perspectives for translating PELNs into clinical and industrial applications.

## 1. Introduction

Plant-derived exosomes (PDEs), also termed plant-derived exosome-like nanoparticles (PELNs), are naturally occurring nanoscale vesicular structures secreted by plant cells through a series of regulated processes including endocytosis, fusion, and exocytosis. These nanoparticles typically exhibit diameters ranging from 30 to 300 nm, characterized by classical phospholipid bilayer membrane structure that closely resembles mammalian exosomes. Their membrane composition shares high homology with plant cell membranes, enabling effective encapsulation and protection of internal bioactive molecules [1]. The lumen of these vesicles contains abundant and diverse functional components, including proteins, lipids, nucleic acids, and plant-specific secondary metabolites, forming the material basis for their biological functions [2]. Functionally, they play critical roles in intercellular communication, immune defense, and stress responses within plants.

Strikingly, recent studies have revealed their cross-kingdom regulatory capabilities, demonstrating multiple biomedical functions in mammalian systems, including drug delivery, anti-inflammatory effects, antitumor activities, tissue repair, and immunomodulation [3]. For instance, PELNs were explored to inhibit inflammatory factor expression, thereby exerting significant anti-inflammatory effects. Moreover, they could also induce cancer cell apoptosis or suppress tumor angiogenesis through carrying specific miRNAs or drug molecules, demonstrating potential antitumor activity [4]. Additionally, they could promote proliferation and differentiation of damaged tissue cells, participate in wound healing processes, and improve pathological conditions such as diabetic ulcers, osteoporosis, and skin aging [5].

Compared with traditional plant extracts, PELNs can retain and deliver intact bioactive components in situ. Due to their natural presence in plant cell walls and intercellular spaces, they are able to avoid degradation or structural alterations in active constituents during conventional extraction processes involving high temperatures, acidic/alkaline conditions, or organic solvents [6]. This “native” characteristic confers higher bioavailability and lower immunogenicity, allowing stable circulation in vivo and efficient traversal across biological barriers for targeted tissue delivery [7].

**Scheme 1:**
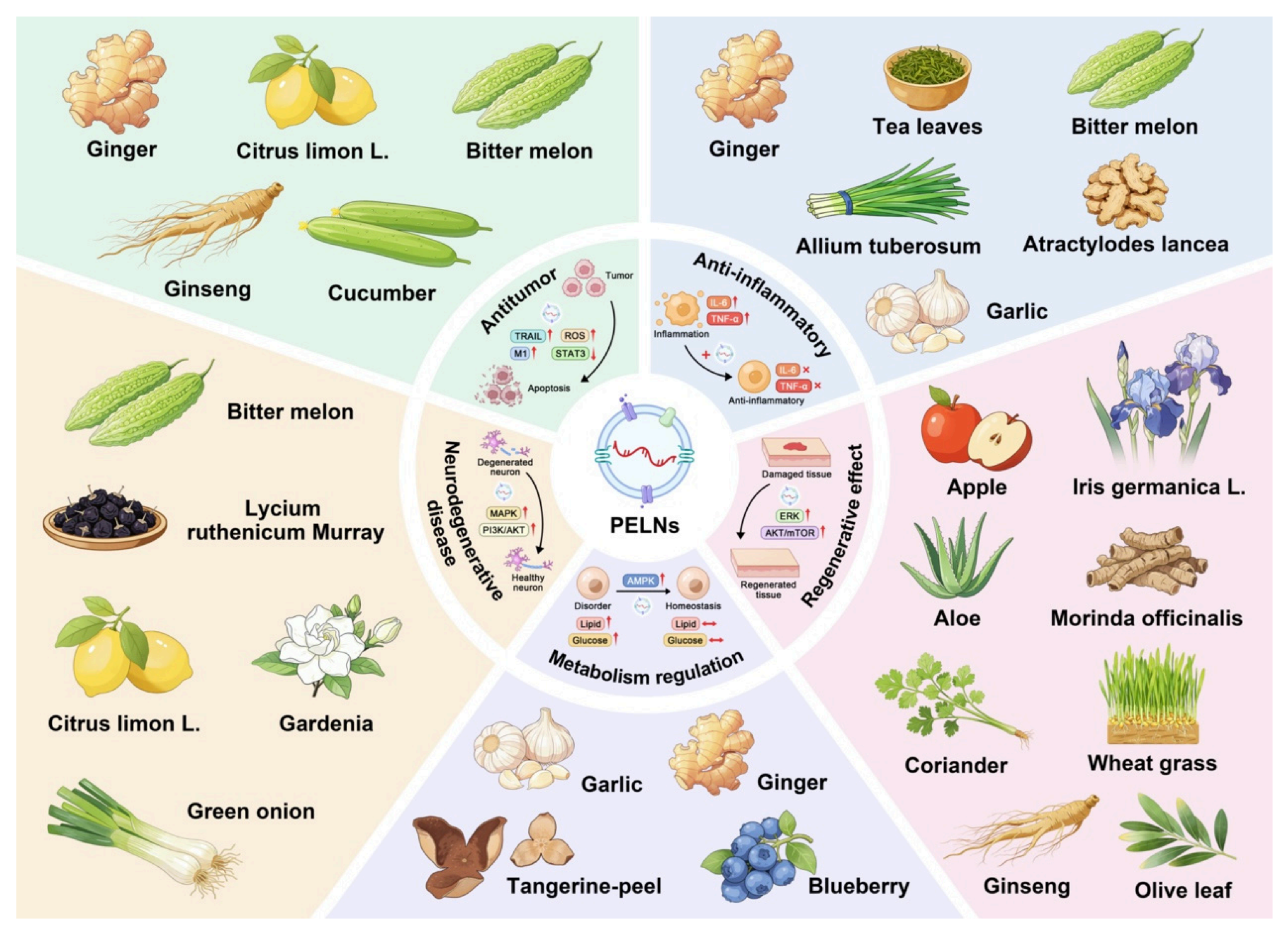
Schematic illustration of the sources and therapeutic potentials of PELNs.

However, translating this natural nanomedicine platform into clinical applications still faces multiple challenges, including establishing standardized extraction and purification protocols for batch consistency, overcoming technical bottlenecks in large-scale production, and elucidating their metabolic pathways and long-term safety profiles in vivo [8]. With advancements in engineering technologies, PELNs hold promise as natural, low-toxicity, high-efficiency drug carriers or standalone therapeutic agents in precision medicine. In this review, we comprehensively discuss PELNs, covering their biogenesis, composition, isolation techniques, biomedical applications, engineering strategies, current challenges, and future perspectives (Scheme 1).

## 2. Biogenesis and Composition

### 2.1. The Process of Biogenesis

The biogenesis of plant exosomes primarily involves three pathways: the multivesicular body (MVB) pathway, the vacuolar pathway, and the exocyst-positive organelle (EXPO) pathway [9]. The MVB pathway is thought to be the basis of PELN biosynthesis, which closely resembles mammalian exosome biogenesis. This process begins with the inward budding of the plasma membrane forming early endosomes that mature into MVBs containing cargo-loaded intraluminal vesicles (ILVs) such as RNA, DNA and lipids. The MVB can either fuse with lysosomes for degradation or escape this fate by fusing with the plasma membrane [10]. Finally, the release of ILVs into the extracellular space as PELNs occurs upon fusion of MVBs with the plasma membrane, a process controlled by cytoskeletal components and Rab proteins [11].

The vacuolar pathway is an integral part of the plant cell’s defense mechanism. When the pathogen invades, intracellular receptors detect danger signals and initiate second messenger responses, such as calcium ion fluxes. This activation prompts SNARE proteins (e.g., VTI11) situated on the tonoplast to facilitate fusion between the vacuole and plasma membrane. Consequently, this not only results in the release of stored antimicrobial peptides and hydrolytic enzymes but also leads to the leakage of cellular contents, causing necrosis. This forms a physical barrier that impedes pathogen dissemination and directly disrupts the cell wall structure of invading organisms [12].

The EXPO pathway is a unique secretory mechanism in plant cells that involves double-membrane EXPO vesicles. Unlike the conventional Golgi secretion and MVB fusion pathways, this pathway directly transports encapsulated cargo into the intercellular space, specifically the cell wall, through targeted fusion of EXPO vesicles with the plasma membrane. This mechanism plays a crucial role in intercellular communication and the transmission of defense signals [13].

### 2.2. The Composition of PELNs

#### 2.2.1. Proteins

Proteomic characterization of plant-derived extracellular vesicles (EVs) has revealed their unique biological properties. The protein composition of these EVs is primarily composed of membrane proteins (e.g., PEN1, PEN3), cytoplasmic proteins, enzymes, and transmembrane proteins, which constitute the structural basis and functional core of these vesicles together [14]. Remarkably, the types and abundance of proteins exhibit significant species-specific characteristics, with an overall lower protein abundance compared to mammalian-derived EVs. These proteins not only serve as biomarkers for identifying the origin of vesicles but also actively participate in important biological processes such as vesicle biogenesis, membrane fusion, and immune regulation, providing a molecular basis for the cross-species bioactivity of plant EVs. For example, PEN1 and PEN3 have been identified as key surface proteins on plant EVs that are involved in plant defense responses [15]. Furthermore, studies have shown that proteins within plant EVs may possess antioxidant capabilities to alleviate disease symptoms [10]. The diverse functions of these proteins underscore the great therapeutic potential of plant EVs in disease treatment and drug delivery applications.

#### 2.2.2. Lipids

The lipid bilayer of PELNs is primarily composed of phospholipids, glycolipids, sterols, and sphingolipids, which constitute the molecular basis for their functional characteristics [16]. The lipid composition directly determines the physicochemical properties of vesicles, thereby influencing their stability, fusion capacity with target cell membranes, and subsequent cellular uptake efficiency [17]. For instance, sterols (e.g., stigmasterol) enhance membrane rigidity, while specific phospholipids may function as signaling molecules to facilitate recognition and binding with particular cell types. Consequently, variations in lipid profiles not only serve as “fingerprints” indicating plant origins but also represent the molecular foundation for cross-species bioactivity and targeted delivery potential. For example, PELNs derived from ginger and turmeric exhibit high abundance of phosphatidic acid, accounting for 35.2% and 34.4% of total lipid content respectively [18], whereas garlic-derived extracellular vesicles are predominantly enriched in free fatty acids, particularly unsaturated fatty acids such as oleic acid and linoleic acid, which demonstrate significant anti-inflammatory and antioxidant bioactivities [19].

#### 2.2.3. Nucleic Acids

Nucleic acids, the fundamental carriers of life’s information, comprise not only deoxyribonucleic acid (DNA) but also a variety of ribonucleic acid (RNA) molecules derived from DNA, including messenger RNA (mRNA), microRNA (miRNA), small interfering RNA (siRNA), and numerous non-coding RNAs, all of which play pivotal roles in regulating gene expression [20]. Among these, miRNAs exhibit significant cross-species gene regulatory functions attributing to their highly conserved sequences across different species. They predominantly act by base complementary pairing, binding specifically to target mRNA molecules at their 3′ untranslated regions (3′UTRs). This interaction results in either the degradation or translational repression of the target mRNA, thereby precisely modulating gene expression levels. Such meticulous regulation is deeply involved in and significantly impacts many crucial biological processes. Notably, it also influences the initiation and equilibrium of immune responses, the evolution and progression of inflammation, and the dysregulated signaling pathways associated with cancer—encompassing cell proliferation, apoptosis, and metastasis. Therefore, miRNAs emerge as vital therapeutic targets in disease research and treatment [21,22].

#### 2.2.4. Secondary Metabolites

In addition to macromolecules, PELNs can also deliver bioactive secondary metabolites such as 6-gingerol, curcumin, sulforaphane, polyphenols and ginsenosides. These compounds enhance their anti-inflammatory, antioxidant and anticancer properties by modulating signaling pathways including AMPK and NF-κB [23]. Meanwhile, specific PELNs such as those derived from broccoli, ginger and Pueraria root have targeted therapeutic effects against gastrointestinal disorders and immune regulation. Studies further revealed that encapsulating metabolites in PELNs (e.g., rutin and β-glucan) promoted cellular uptake and accumulation, with the concentration of metabolites varying among different density fractions of PELNs, suggesting the specificity potential of loading capacity [24].

## 3. Separation and Characterization Techniques

### 3.1. Separation Techniques

The extraction of PELNs necessitates a meticulous preprocessing of the plant tissues. This involves washing and grinding procedures designed to release juice or interstitial fluid abundant in EVs. The subsequent purification process demands an integrated application of techniques in a systematic sequence such as differential centrifugation, density gradient centrifugation, size-exclusion chromatography and so on.

Ultracentrifugation is the most commonly used method. Fresh plant tissues are first homogenized at high speed and filtered, followed by low-speed centrifugation to remove coarse particles. After obtaining the supernatant, a series of rate-increasing centrifugations were performed, and finally ultracentrifugation was performed at 100,000–120,000× *g* for 90 min to pellet exosomes, which were then resuspended in PBS buffer [25]. This method is considered as “gold standard” due to its simple procedure and low risk of contamination which has been successfully applied to extract exosomes from various plants and validate their therapeutic potential in disease models [26]. However, this technique also has significant limitations. Firstly, repeated ultracentrifugation can damage the integrity and biological activity of exosomes. Secondly, stringent conditions, special requirements, and high cost associated with the procedure limit its applicability. Moreover, impurities such as fibers and starch in plant juices often result in insufficient purity, while additional centrifugation steps aimed at improving purity further reduce yield [27].

Density gradient centrifugation is a pivotal technique for the purification of PELNs. The procedure involves creating a density gradient liquid column using media such as sucrose or iodixanol, with concentrations stratified at 8%, 30%, 45%, and 60% from top to bottom. After positioning the preliminarily centrifuged sample atop this gradient, extended ultracentrifugation allows PELNs to sediment into layers that match their buoyant densities, typically ranging between 1.13 and 1.19 g/mL, which corresponds to the 30–45% sucrose interfaces. Subsequently, the desired fraction is harvested to yield highly pure extracellular vesicles [28]. This approach markedly improves the purity of PELNs while preserving their structural integrity, rendering it especially apt for research necessitating high purity levels [29]. Nonetheless, the resulting yield is limited by the intricacies and extended duration of this method, and its potential inability to entirely eliminate prevalent macromolecular contaminants from plants restrict its large-scale deployment [30].

Ultrafiltration is an effective method for size-based separation of extracellular vesicles that uses a membrane with specific pore sizes to capture large particles (>200 nm) and pass through small particles (<20 nm). This process often involves applying pressure or low-speed centrifugation to speed up sample processing, making it suitable for handling large volumes of samples while maintaining the activity of the vesicles [31]. The advantages of this method include ease of operation, fast processing time, no need for special equipment, and the ability to select vesicles of specific sizes by selecting the pore size of the membrane. Therefore, its use in research and therapeutic development has been increasing, with centrifugal ultrafiltration being considered a superior approach. However, ultrafiltration can be limited in efficiency due to clogging of the membrane or trapping of the vesicles, and it cannot completely remove minute impurities. Thus, it is often necessary to combine it with other techniques such as differential centrifugation to improve purity. When using this method, it is important to pretreat the sample to remove large membrane structures. In addition, the centrifugal force and time should be carefully controlled to prevent membrane breakage and ensure proper separation [32].

Size-exclusion chromatography (SEC) is a separation technique based on the hydrodynamic volume of particles, which separates PELNs from other biomolecules in samples by size using porous gel or resin columns [33]. This method allows for efficient isolation of vesicles with high purity, structural integrity, and preserved bioactivity, making it particularly suitable for downstream functional and compositional analyses. SEC is often combined with differential centrifugation, ultrafiltration, and other techniques to further improve purity and yield. Its mild processing conditions and scalability make it suitable for large-scale preparations [34]. Although conventional SEC suffers from operational complexity, sample dilution, time consumption, and difficulty in completely removing impurities of similar sizes, the advent of commercialized chromatography columns has enabled rapid, standardized extraction with high purity and reusability advantages, establishing it as one of the mainstream separation methods currently available [35].

The polymer precipitation method is a common technique for isolating PELNs using hydrophilic polymers such as polyethylene glycol (PEG). The principle involves the binding of PEG with water molecules to form a hydrophobic microenvironment around vesicles, thereby reducing their solubility and inducing aggregation. These aggregated vesicles can then be precipitated and collected by low-speed centrifugation. This method is widely used due to its simplicity, low cost, speed, minimal damage to vesicles, and scalability for large-scale production. However, its main drawback is the relatively low purity, as PEG also precipitates impurities such as proteins [36]. To improve purity, PEG precipitation is often combined with differential centrifugation, filtration, or other techniques, or a two-phase system of PEG/dextran is adopted to reduce co-precipitation of contaminants such as proteins. Optimized methods, such as using specific concentrations of PEG 6000, pectinase, and sodium chloride, have successfully isolated nanovesicles with good bioactivity and intact morphology from various plant sources. These nanovesicles have shown potential applications in functional studies, such as skin repair [37].

Microfluidics is a miniaturized platform that uses acoustic wave, electrophoresis and fluid dynamics principles to efficiently separate PELNs based on their physical and biochemical properties in microchannels [38]. It integrates separation, recovery and detection processes with the advantages of miniaturization, automation, high-throughput capacity, minimal sample consumption and significantly shortened processing time [39]. However, current practical applications still face challenges such as limited sample throughput, insufficient processing capacity, operational complexity and lack of standardization, and related research is still in its early stages [26]. Future development requires further optimization of equipment compatibility, stability and versatility to promote its application in high-throughput analysis and clinical transformation of plant-derived extracellular vesicles.

Other methods include aqueous two-phase systems, immunoaffinity capture, and electrophoresis-dialysis technologies. However, these methods have challenges with yield and purity trade-offs and may exhibit batch-to-batch variations (Table 1).

### 3.2. Characterization Techniques

#### 3.2.1. Morphology and Size Analysis

Transmission electron microscopy (TEM) is a critical method for evaluating nanovesicle ultrastructure and the most widely used technique to obtain images of PELNs with nanoscale resolution, allowing observation of internal structure and morphology [40]. However, TEM is not only time-consuming but may also cause vesicle deformation due to dehydration during sample preparation, leading to cup-shaped artifacts. In contrast, cryo-electron microscopy (Cryo-EM), which observes rapidly frozen particles at low temperatures, effectively avoids dehydration issues and better preserves the spherical morphology of vesicles [41]. Scanning electron microscopy (SEM) is mainly used to investigate surface morphology, size distribution, and structural details of vesicles [42]. In addition, atomic force microscopy (AFM) provides high-resolution three-dimensional visualization of membranes and enables quantification of adhesion, elasticity, and deformation properties, although its testing process is complex and imposes stringent requirements on samples [43]. These techniques together form the core toolkit for morphological studies of plant-derived extracellular nanovesicles.

#### 3.2.2. Particle Size and Potential Analysis

The particle size distribution can be calculated with dynamic light scattering (DLS) by measuring the fluctuation in scattered light when a monochromatic light source irradiates the sample. This technique is highly sensitive and allows for rapid evaluation of PELN size and charge with minimal sample volume, but has relatively low resolution [44]. Zeta potential, as an important parameter for evaluating colloidal stability, reflects the aggregation tendency and stability of vesicles [45,46]. The combination of these two techniques can comprehensively characterize the physical properties of plant-derived extracellular vesicles, providing basic data for subsequent functional studies.

#### 3.2.3. Biochemical Composition Analysis

Mass spectrometry techniques such as liquid chromatography–tandem mass spectrometry can accurately identify not only the types and abundance of proteins but also lipid molecules in PELNs, thereby clarifying their structural basis and sources of biological activity. For example, proteomic analysis can reveal membrane channel proteins, cytoskeletal proteins, and signaling proteins that are closely related to vesicle targeting and cellular uptake mechanisms [47]. Moreover, high-throughput sequencing technology focuses on nucleic acid analysis, comprehensively deciphering the type and expression profile of small RNA carried by exosomes, including miRNA, mRNA, and lncRNA [48]. These nucleic acid molecules act as important messengers for cross-species gene regulation, and their identification is essential to understand the mechanism by which plant exosomes regulate gut microbiota, immune response, or disease pathways. The combination of these two technologies provides a powerful tool for decoding the complex life activities of plant exosomes at the molecular level.

#### 3.2.4. Purity Assessment

Purity assessment is a crucial step to ensure the reliability of PELN research, which is usually performed by detecting specific protein markers and potential contaminants. At present, research on specific markers for PELNs is still insufficient. However, several candidate proteins such as PEN1 and TET8 have been identified as potential markers for identifying plant origin [49]. Meanwhile, techniques including Western blotting and flow cytometry are commonly used to verify the presence of these markers. On the other hand, impurity detection mainly focuses on co-isolated proteins, nucleic acids or residual polymers. For example, PEG residues need to be detected in PEG precipitation methods, while macromolecular protein contamination levels need to be evaluated after ultrafiltration procedures [50]. By comprehensively evaluating both the enrichment efficiency of specific markers and the removal effect of impurities, the overall purity of PELN preparation can be systematically determined, thereby laying a foundational basis for subsequent functional investigations and applications.

## 4. Biomedical Applications

As the high biocompatibility, low immunogenicity and efficient targeting capabilities of PELNs, they have shown great promise in the treatment of numerous diseases, including cancer, inflammation, tissue repair, metabolism regelation and neurodegenerative protection.

### 4.1. Antitumor Activity of PELNS

**Figure 1:**
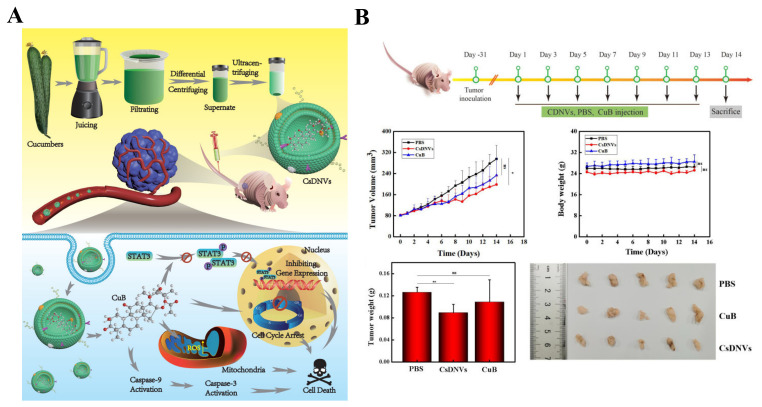
(**A**) Schematic illustration and mechanism of CDNVs in antitumor treatment. (**B**) The antitumor properties of CDNVs in vivo, including tumor volume, mice body weight, and tumor weight. Obtained from Ref. [54]. Copyright © 2022, Tingting Chen et al. * *p* < 0.05 and ** *p* < 0.01.

PELNs could exert antitumor effects by a variety of mechanisms. Firstly, they could directly induce tumor cell apoptosis, cell cycle arrest, and metastasis inhibition. Researchers have found that exosome-like nanoparticles derived from ginger (GELNs) selectively inhibited the growth of aggressive triple-negative breast cancer (TNBC) cells through the induction of apoptosis, generation of reactive oxygen species (ROS), and cell cycle arrest. Additionally, GELNs also impeded cancer cell migration and colony formation in vitro [51]. PELNs isolated from lemon juice (*Citrus limon* L.) were identified to exhibit anticancer properties by triggering TRAIL-mediated apoptosis in tumor cells. In vitro experiments showed these vesicles inhibit cancer cell proliferation across multiple lines, while in vivo tests demonstrated their ability to target tumors and suppress chronic myeloid leukemia (CML) growth through the same apoptotic pathway. This is the first report of lemon nanovesicles with such specific antitumor activity [52]. Similarly, *Citrus limon* L.-derived nanovesicles were investigated as potential agents for colorectal cancer (CRC) prevention, particularly in cases involving p53 inactivation. The nanovesicles inhibited cell growth in both p53-wild and p53-inactivated CRC cells, with uptake mediated by micropinocytosis which was a pathway activated by p53 inactivation. Although the nanovesicles contained citrate, their growth-inhibitory effects were independent of p53 status, suggesting a novel mechanism for CRC prevention via micropinocytosis [53]. Cucumber-derived nanovesicles (CDNVs) was demonstrated to have potent anticancer effects in vitro and in vivo by inhibiting STAT3 activation, inducing ROS, arresting cell cycles, and activating caspases. Notably, CDNVs reduced tumor growth in mice without organ toxicity, highlighting their safety profile [54] (Figure 1).

**Figure 2:**
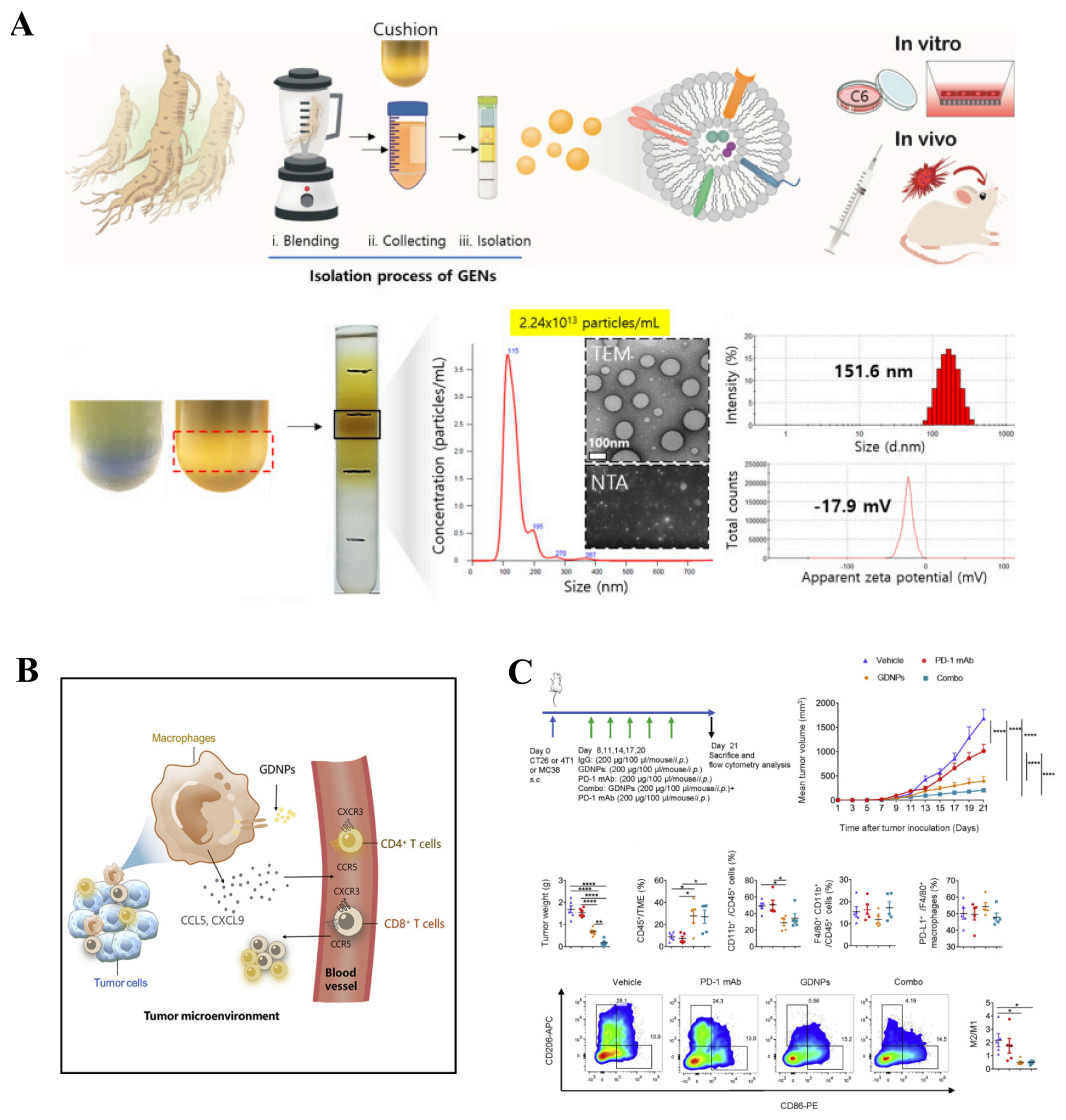
(**A**) Schematic illustration of the isolation process and characterization of GENs from fresh ginseng. Obtained from Ref. [56]. Copyright © 2023, Jisu Kim et al. (**B**) The mechanism of GDNPs in potentiating immune checkpoint antibody efficacy by reprogramming the cold tumor microenvironment. (**C**) Combination therapy using GDNPs and PD-1 mAb elicited rejection of CT26 murine colon tumor by polarizing M2-like macrophares to M1-like phenotype. Obtained from Ref. [57]. Copyright © 2022, Xuan Han et al. * *p* < 0.05, ** *p* < 0.01 and **** *p* < 0.001.

Secondly, PELNs could elicit antitumor immune response by modulating the tumor microenvironment. Ginseng-derived nanoparticles (GDNPs) were found to effectively reprogram tumor-associated macrophages from protumor M2 to antitumor M1 phenotype. This polarization was dependent on TLR4/MyD88 signaling pathway activation and resulted in significant tumor suppression in melanoma-bearing mice. The immunomodulatory effect of GDNPs was attributed to both the ceramide lipids and proteins present in the nanoparticles [55]. In addition, ginseng-derived exosome-like nanoparticles (GENs) could also effectively cross the blood–brain barrier and target glioma. It showed that GENs reshaped the tumor immune microenvironment by significantly increasing the infiltration of M1-type tumor-associated macrophages, thereby inhibiting tumor growth. Experiments demonstrated that GENs effectively regulated immune responses and delay glioma progression in both in vitro and in vivo models [56]. Moreover, GDNPs and anti-PD-1 antibody was developed a combination therapy to overcome the resistance of “cold” tumors to immunotherapy. GDNPs effectively reprogrammed tumor-associated macrophages, which secreted chemokines that recruited CD8^+^ T cells into the tumor microenvironment. The conversion from a “cold” state to a “hot” state synergized with PD-1 blockade to significantly enhance antitumor immunity in multiple murine models without detectable systemic toxicity [57] (Figure 2).

Thirdly, some PELNs could improve drug sensitization in antitumor treatment. Yang et al. found that bitter melon-derived extracellular vesicles (BMEVs) exhibited potent anticancer effects against oral squamous cell carcinoma (OSCC) and effectively reverse resistance to the chemotherapeutic agent 5-FU. Mechanistically, BMEVs significantly induced apoptosis in cancer cells via ROS generation and c-Jun upregulation, while concomitantly suppressing NLRP3 inflammasome activation which was identified as the critical mechanism underlying the attenuation of OSCC cell resistance to 5-FU in vitro [58].

In addition, grapefruit-derived exosome-like nanovesicles (ELPDNVs) contain measurable levels of ascorbic acid and other antioxidant molecules. These ELPDNVs inhibited the growth of human leukemic cells and patient-derived bone marrow blasts without affecting normal cells. The antiproliferative effect of ELPDNVs on leukemic cell lines was comparable to that of high-dose ascorbic acid. Additionally, ELPDNVs increased reactive oxygen species levels in leukemic cells but not in normal cells, similar to ascorbic acid [59].

### 4.2. Anti-Inflammatory Activity of PELNS

PELNs could effectively alleviate inflammatory diseases by inhibiting related signaling pathway, regulating inflammatory factors and promoting the expression of anti-inflammatory cytokines.

**Figure 3:**
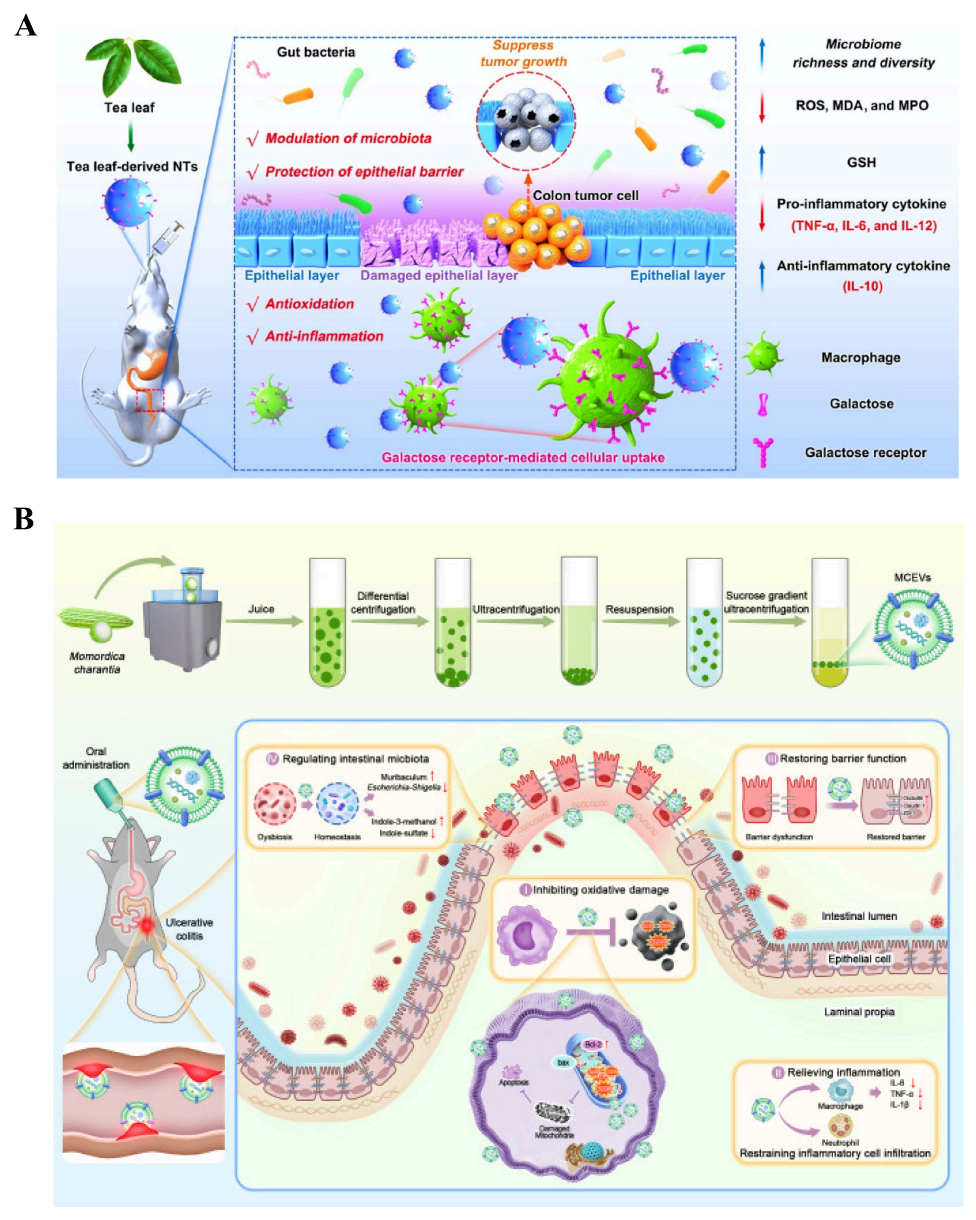
(**A**) Schematic illustration of the tea leaf-derived natural NTs for orally targeted prevention and alleviation of colon diseases. Obtained from Ref. [61]. Copyright © 2021, Menghang Zu et al. (**B**) Schematic illustration of oral administration of MCEVs for treating ulcerative colitis through multiple synergistic pathways. Obtained from Ref. [62]. Copyright © 2025, Bowen Gao et al.

Researchers found 27 miRNAs enriched in ginger-derived exosome-like nanoparticles (GELNs), which are linked to anti-inflammatory and anticancer pathways. These nanoparticles were internalized by intestinal cells via caveolin-mediated endocytosis and micropinocytosis, reducing LPS-induced inflammation by suppressing NF-κβ, IL-6, IL-8, and TNF-α. The therapeutic effects were attributed to GELN-carried miRNAs, suggesting cross-kingdom regulatory potential in vitro [60]. In addition, Xiao’s group have developed natural exosome-like nanotherapeutics (NTs) from tea leaves that are efficiently taken up by macrophages via galactose receptors. These NTs effectively reduced inflammation and oxidative stress while promoting anti-inflammatory responses in the colon. After oral administration, they could restore intestinal barrier function and improve gut microbiota diversity, demonstrating strong therapeutic potential against inflammatory bowel disease and colitis-associated cancer in vivo [61]. Extracellular vesicles derived from *Momordica charantia* (MCEVs) exhibited significant anti-inflammatory and antioxidant properties by safeguarding cells against oxidative stress and inhibiting macrophage inflammation. In vivo investigations revealed that MCEVs preferentially accumulated in inflamed colon tissues, ameliorating ulcerative colitis by modulating the intestinal microenvironment [62] (Figure 3). Garlic-derived exosome-like nanoparticles (GELNs), rich in proteins and microRNAs, showed promise in treating ulcerative colitis (UC) by stabilizing gut health. In mice with colitis, oral administration of GELNs alleviated symptoms like diarrhea, reduced inflammation, and repaired the gut barrier. The results demonstrated that specific microRNA (peu-MIR2916-p3) in GELNs promoted growth of beneficial bacterium which was linked to colitis relief [63].

In neurological disorders, exosome-like nanoparticles derived from Allium tuberosum (A-ELNs) significantly attenuated neuroinflammation by suppressing nitric oxide production and pro-inflammatory cytokine expression in lipopolysaccharide (LPS)-activated microglial cells. Mechanistic analysis revealed this anti-neuroinflammatory effect was mediated through transcriptional modulation of key inflammatory regulators, characterized by upregulation of heme oxygenase-1 and downregulation of inducible nitric oxide synthase. Notably, when functionalized with dexamethasone (Dex-A-ELNs), these nanoparticles exhibited synergistic therapeutic enhancement compared to individual treatments in vivo [64]. The exosome-like nanoparticles isolated from Atractylodes lancea (ALR-ELNs) were found to effectively inhibit lipopolysaccharide (LPS)-induced neuroinflammation. The ALR-ELNs were demonstrated to decrease the production of pro-inflammatory mediators including NO, IL-1β, IL-6, and TNF-α at both molecular and protein levels. These results indicated that ALR-ELNs have distinct anti-neuroinflammatory activities in microglial cells via multitarget modulation of inflammatory factors in vitro [65].

### 4.3. Regenerative Effect of PELNS

The application of PELNs in regenerative medicine is gradually becoming a research hotspot, with their unique advantages and potential applications as follows:

Firstly, PELNs were certified to apply in the treatment of hair loss as they could increase hair density and thickness by modulating scalp microbiota and promoting hair follicle growth. Kim’s group found that exosomes derived from *Iris germanica* L. effectively protected human hair follicle cells against oxidative stress and mitochondrial damage induced by dihydrotestosterone. These exosomes significantly decreased ROS, restored mitochondrial function, and improved key cellular activities such as cell migration and the formation of follicle-like spheroids. Furthermore, the exosomes were shown to activate the Wnt/β-catenin pathway via GSK-3β, AKT, and ERK signaling, leading to β-catenin stabilization and the expression of genes associated with hair growth in vitro [66].

**Figure 4:**
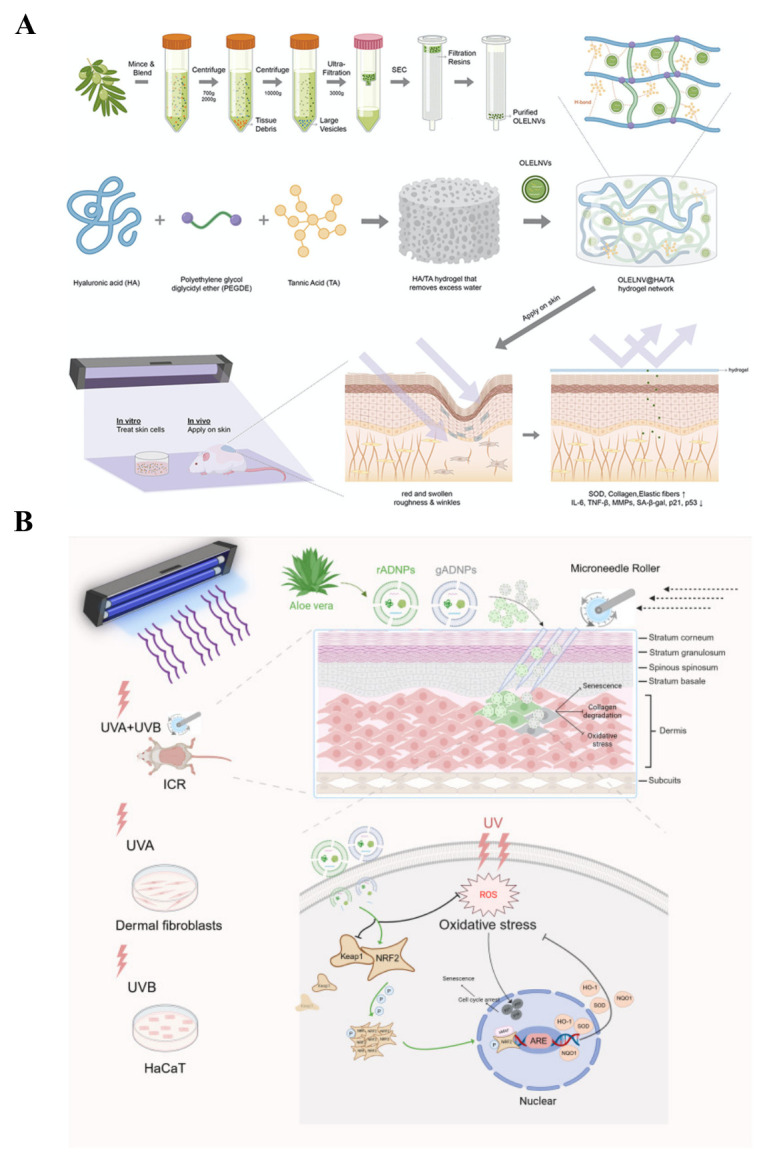
(**A**) Schematic illustration of the dual-action hydrogel combined with OLELNVs to combat photoaging caused by UV exposure. Obtained from Ref. [68]. Copyright © 2024, Zhenzhen Wang et al. (**B**) The mechanism of ADNPs in reducing UV-induced oxidative stress, DNA damage, and aging markers by activating the Nrf2/ARE pathway for anti-photoaging therapy. Obtained from Ref. [69]. Copyright © 2025, Zixuan Sun et al.

Secondly, PELNs are widely used in anti-aging. Apple-derived nanovesicles (ADNVs) functioned as a unique anti-inflammatory agent to counteract skin aging by inhibiting the TLR4 receptor and the subsequent NF-κB pro-inflammatory pathway. Additionally, ADNVs contributed to skin rejuvenation by promoting collagen synthesis and diminishing the production of matrix-degrading metalloproteinases in vivo [67]. A dual-action hydrogel dressing which combined olive leaf-derived nanovesicles (OLELNVs) to combat photoaging caused by UV exposure. Results showed the system effectively reduced UV-induced skin damage and promotes repair, while RNA-seq analysis linked its efficacy to suppression of the NF-κB pathway, mitigating inflammatory aging. The innovation addressed gaps in current anti-photoaging strategies by integrating prevention and repair in vitro [68]. Furthermore, aloe-derived nanoparticles (ADNPs) from gel and rind was developed as a novel anti-photoaging therapy in reducing UV-induced oxidative stress, DNA damage, and aging markers by activating the Nrf2/ARE pathway. In vivo experiments showed that ADNPs could decrease oxidative markers in mice and delay skin aging with high biocompatibility [69] (Figure 4).

**Figure 5:**
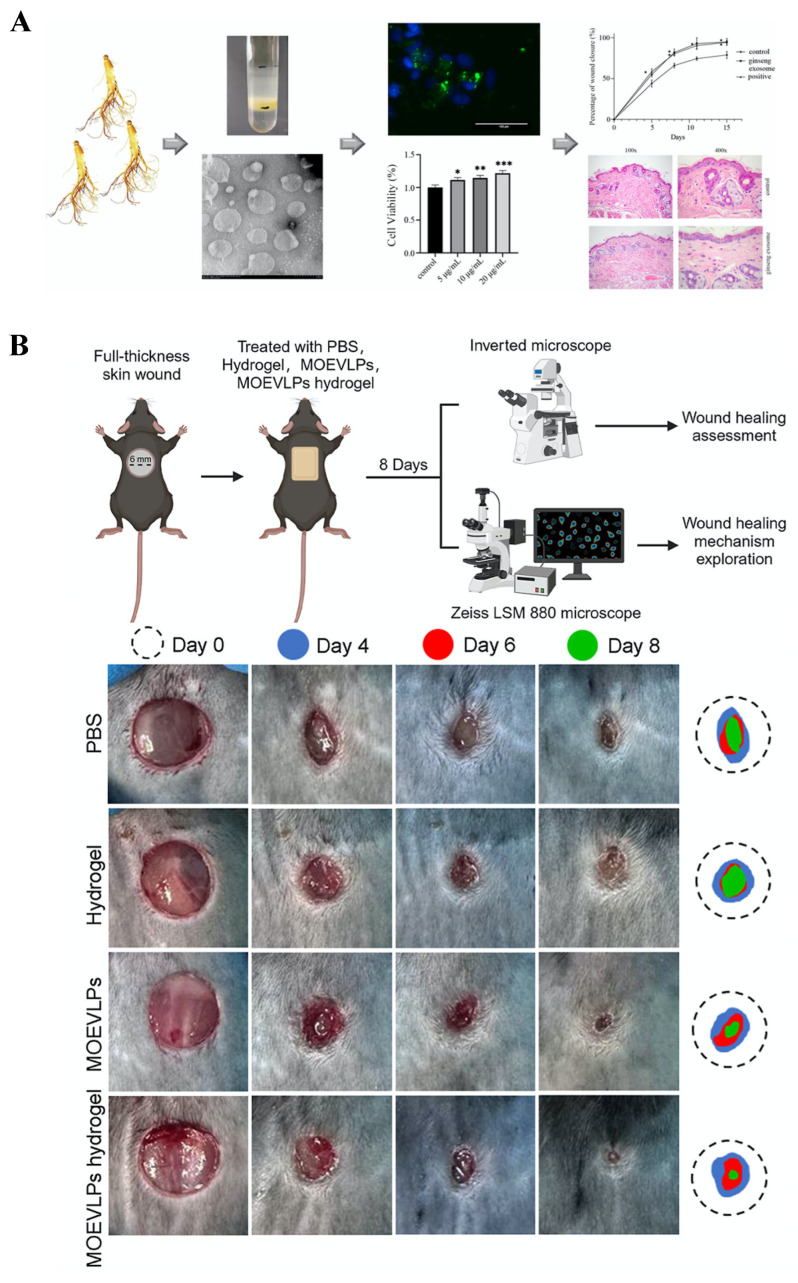
(**A**) Schematic illustration of GDNPs in inducing skin cell proliferation and promote wound healing. Obtained from Ref. [70]. Copyright © 2023, Song Yang et al. (**B**) The representative images of the temporal progression of wound healing following MOVELP treatment from day 0 to 8 in vivo. Obtained from Ref. [73]. Copyright © 2025, Qing Zhao et al. * *p* < 0.05, ** *p* < 0.01 and *** *p* < 0.005.

Thirdly, PELNs were verified to promote cell migration and tissue repair. Researchers found that ginseng-derived nanoparticles (GDNPs) significantly promoted skin wound healing by enhancing the proliferation and migration of keratinocytes, fibroblasts, and endothelial cells. GDNPs were shown to upregulate the secretion of essential extracellular matrix components and stimulate angiogenesis via activation of the ERK and AKT/mTOR signaling pathways. In murine models, GDNPs effectively accelerated wound closure and reduced inflammation, suggesting their potential as an innovative therapeutic option for chronic wound management [70]. In addition, exosome-like nanovesicles derived from coriander (CDENs) was identified to effectively facilitate wound healing by enhancing cell migration, neutralizing ROS, and mitigating inflammation. The hydrogel based on CDENs exhibited sustained release effect and significantly expedited wound healing in vivo by fostering macrophage M2 polarization, angiogenesis, and collagen deposition throughout various stages of healing [71]. Moreover, PELNs derived from wheat grass significantly accelerated wound healing through enhanced cell viability and migration across multiple cell types. These plant-derived exosomes were shown to promote cellular proliferation, upregulate collagen synthesis, inhibit apoptosis, and stimulate angiogenesis in vitro [72]. Furthermore, extracellular vesicle-like particles isolated from Morinda Officinalis (MOEVLPs) could effectively promote endothelial cell proliferation, migration, and tube formation in vitro. These MOEVLPs were demonstrated to activate the MAPK/YAP1 signaling pathway and were subsequently incorporated into a hydrogel carrier to prolong their release in vivo. The results showed that MOEVLP-loaded hydrogel significantly enhanced angiogenesis and accelerated wound healing in a full-thickness skin wound model, which presented a potent hydrogel-based strategy utilizing plant-derived vesicles for advanced wound care [73] (Figure 5). Organic plant-derived extracellular vesicles (PDEVs) contain high levels of antioxidants and show increased antioxidant power compared to PDEVs from single plants, suggesting a synergistic effect. A mixture of PDEVs from five fruits contained detectable levels of citric acid, ascorbic acid, glutathione, catalase, and SOD. When applied to H_2_O_2_-conditioned human fibroblasts, the PDEV mixture reversed redox imbalance, restored mitochondrial homeostasis, and reduced mitochondrial superoxide while increasing sirtuin levels. The antioxidant action also promoted wound repair in a fibroblast monolayer, associated with increased vimentin and matrix metalloproteinase-9 expression [74].

### 4.4. Metabolism Regulation of PELNS

PELNs were explored to play an important role in metabolism regulation, including glucose and lipid metabolism. Garlic-derived exosome-like nanoparticles (GaELNs) were found to alleviate obesity and associate brain inflammation in mice. Research demonstrated that specific lipid in GaELNs binds to BASP1 in brain microglial cells, which inhibited the c-Myc/STING signaling pathway and reduced the production of inflammatory cytokines. This treatment also improved glucose metabolism, insulin sensitivity, and memory function in the mice. The study revealed that dietary nanoparticles could reverse the detrimental effects of a high-fat diet by targeting brain inflammation through specific molecular mechanism [75]. Similarly, Zhang’s group discovered that gut bacteria could be trained by diet-derived nanoparticles to release beneficial outer membrane vesicles (OMVs). They found that OMVs from garlic nanoparticle-trained Akkermansia muciniphila could reverse type 2 diabetes and reduced brain inflammation in mice. These OMVs worked by increasing specific proteins and lipids that enhanced anti-diabetic hormones and suppressed inflammatory pathways [76]. Moreover, ginger-derived nanoparticles (GDNP) were also discovered to prevent high-fat diet-induced insulin resistance and obesity in mice. They found that GDNP could protect Foxa2 protein in gut epithelial cells from inactivation, which in turn altered the composition of intestinal exosomes and prevented insulin resistance [77].

**Figure 6:**
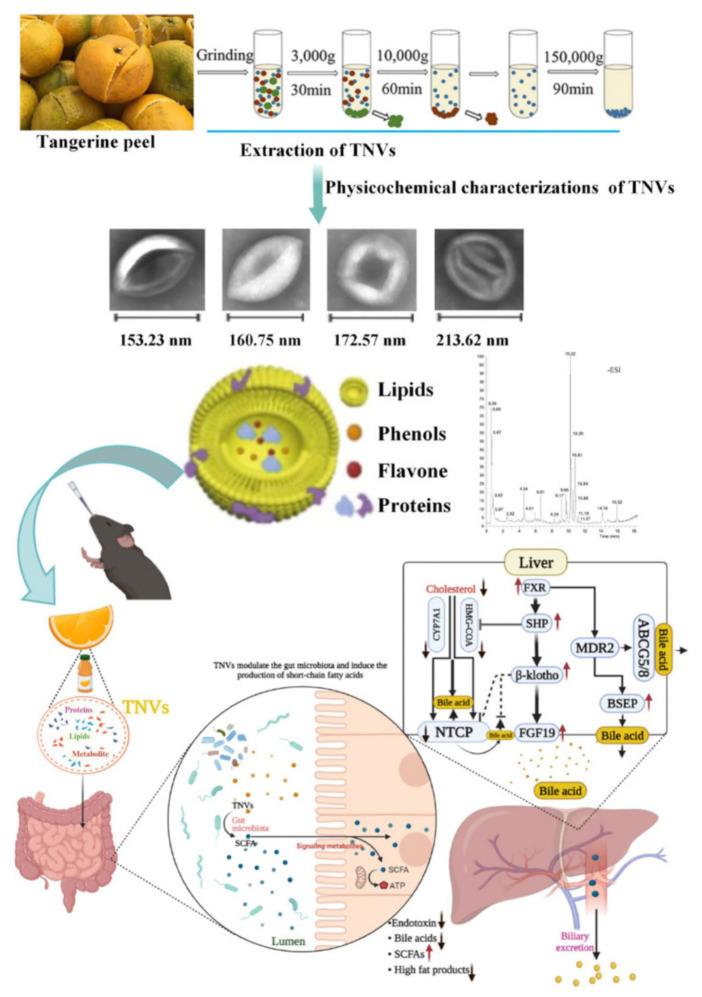
Schematic illustration of TNVs in alleviating hepatic steatosis by regulating lipid metabolism and intestinal microflora. Obtained from Ref. [78]. Copyright © 2024, Junju Zou et al.

In addition, tangerine-peel-derived exosome-like nanovesicles (TNVs) were explored as potential treatment for hepatic lipotoxicity in type 2 diabetes (T2DM). Results showed TNVs reduced insulin resistance, hepatic lipid accumulation, and gut dysbiosis while restoring metabolic gene expression (e.g., AMPK, PPAR-γ) and enhancing fatty acid β-oxidation and glycolysis pathways. Cell experiments confirmed that TNVs could significantly lower lipid accumulation by regulating glucose/lipid metabolism genes. The findings highlighted TNVs as a novel therapeutic candidate for improving glucose and lipid dysregulation in T2DM-associated non-alcoholic fatty liver disease (NAFLD) [78] (Figure 6). Additionally, blueberry-derived exosome-like nanoparticles (BELNs) were verified to alleviate NAFLD by reducing oxidative stress and improving mitochondrial function. Their findings showed that BELNs activated the Nrf2 pathway to enhance antioxidant defenses and decreased ROS in both cellular and mouse models. Moreover, BELNs were found to inhibit key lipid synthesis genes and reduce fat accumulation in liver [79].

### 4.5. Function of PELNS in Neurodegenerative Disease Treatment

PELNs were found to be able to cross the blood–brain barrier and carry neuroprotective agents for the treatment of Alzheimer’s disease, Parkinson’s disease, and stroke. Exosome-like nanoparticles derived from green onions (GDENs) were elucidated to have the capacity to inhibit glutamate-induced ferroptosis in mouse hippocampal cells. The study revealed that GDENs exerted protective effects through multiple mechanisms, including attenuating calcium influx, suppressing lipid peroxidation, reducing iron accumulation, and enhancing endogenous antioxidant defense systems [80]. Moreover, exosome-like nanovesicles isolated from *Citrus limon* (EXO-CLs) were investigated to possess the ability to traverse the blood–brain barrier and offer protection to neuronal cells against oxidative stress in vitro [81]. Moreover, exosome-like nanovesicles from Lycium ruthenicum Murray (LRM-ELNs) were found to effectively inhibit Ab-induced apoptosis in neuronal cells by activating the MAPK and PI3K/AKT signaling pathways in vitro [82].

In addition, the therapeutic potential of Gardenia jasminoides-derived extracellular vesicles (GDEVs) was explored in models of Parkinson’s disease. In rotenone-induced models using PC12 neuronal cells and Caenorhabditis elegans, GDEVs were certified to mitigate mitochondrial dysfunction, decrease cytochrome C release, and reduce α-synuclein accumulation. The vesicles suppressed apoptosis by modulating the p38 MAPK/p53 pathway and elevating the Bcl-2/Bax ratio, thereby safeguarding dopaminergic neurons and enhancing dopamine release in vitro [83].

Furthermore, the neuroprotective potential of exosome-like nanoparticles from *Momordica charantia* (MC-ELNs) were investigated against cerebral ischemia–reperfusion injury. They found that MC-ELNs effectively crossed the blood–brain barrier, reduced infarct size, and preserved blood–brain barrier integrity by modulating proteins like MMP-9, claudin-5, and ZO-1. The study further revealed that MC-ELNs attenuated neuronal apoptosis by upregulating the AKT/GSK3b signaling pathway in vitro [84]. Research progress was summarized as shown in Table 2.

In summary, ginger-derived vesicles exhibit antitumor effects by inducing apoptosis, generating ROS, and causing cell cycle arrest in triple-negative breast cancer cells, while simultaneously alleviating intestinal inflammation and preventing high-fat diet-induced insulin resistance and obesity. Lemon-derived vesicles exhibit antitumor effects by inhibiting the growth cancer cells through TRAIL-mediated apoptosis, and cross the blood–brain barrier to protect neurons from oxidative stress, with potential applications in Alzheimer’s disease. Cucumber-derived vesicles could inhibit STAT3 activation to suppress tumor growth in vivo without organ toxicity. Ginseng-derived vesicles exhibit antitumor immunity by polarizing tumor-associated macrophages from the M2 phenotype to the M1 phenotype, thereby enhancing the efficacy of anti-PD-1 therapy and remodeling the cold tumor microenvironment. Moreover, they can also accelerate skin wound healing. Bitter melon-derived vesicles showed antitumor effects against oral squamous cell carcinoma. In terms of anti-inflammatory properties, it preferentially accumulated in inflamed tissues during ulcerative colitis and modulates the gut microenvironment. Regarding neuroprotection, it could crosse the blood–brain barrier to mitigate cerebral ischemia–reperfusion injury while preserving the integrity of the blood–brain barrier. Tea-derived vesicles could alleviate colonic inflammation and oxidative stress, restore intestinal barrier function, improve microbial diversity, and demonstrated therapeutic potential for inflammatory bowel disease (IBD) and colitis-associated cancer. Garlic-derived vesicles was demonstrated to alleviate symptoms of ulcerative colitis, and repair the intestinal barrier. Moreover, it mitigated obesity-associated neuroinflammation and improved glucose metabolism as well as insulin sensitivity. Apple-derived vesicles exhibit anti-skin aging properties by inhibiting the TLR4/NF-κB signaling pathway, promoting collagen synthesis, and reducing matrix metalloproteinase production. Olive leaf and aloe-derived vesicles could attenuate UV-induced skin damage and inflammatory aging. Tangerine peel and blueberry-derived vesicles indicated for non-alcoholic fatty liver disease.

The biological effects of PELNs can be attributed to three major types of bioactive cargo: lipids, proteins, and microRNAs.

1. Effects attributed to microRNAs (RNA cargo): The anti-inflammatory effects of Ginger-derived exosome-like nanoparticles were directly attributed to 27 enriched miRNAs carried by the nanoparticles. The alleviation of ulcerative colitis by Garlic-derived exosome-like nanoparticles was linked to a specific microRNA (peu-MIR2916-p3) that promoted beneficial gut bacteria.

2. Effects attributed to lipids and proteins (combined): Ginseng-derived nanoparticles facilitated the reprogramming of tumor-associated macrophages from M2 to M1 phenotype, which was attributed to both ceramide lipids and proteins present in the nanoparticles.

3. Effects attributed to lipids (alone): The alleviation of obesity and associated brain inflammation was mediated by a specific lipid that binds to the BASP1 protein in brain microglial cells from garlic-derived exosome-like nanoparticles.

## 5. Engineering Strategies of PELNS

### 5.1. Preparation and Characterization of P/M Composite

The conjugation of targeting ligands onto PELNs surfaces via chemical modifications or biological conjugations can significantly enhance their tissue-specific delivery capabilities. These engineering strategies not only increase drug accumulation at diseased sites but also reduce off-target toxicity to normal tissues, representing a key technological approach for achieving precision therapy [85]. For example, grapefruit-derived nanovectors (GNVs) were utilized to develop a drug delivery system to transport therapeutic RNA (miR17) into the brain for tumor treatment. By functionalizing these nanovectors with folic acid, targeted delivery to brain tumor cells was achieved. Intranasal administration of this formulation could effectively deliver miR17 to tumor sites and significantly inhibit tumor growth in murine models [86]. In addition, grapefruit-derived EVs was verified as superior alternatives to mammalian exosomes for drug delivery. Through the engineering of these PELNs with aptamers via click chemistry, specific targeting capabilities were achieved, resulting in significantly enhanced cellular uptake in brain cells. This functionalization method yielded cost-effective and mass-producible carriers that exhibited comparable efficacy to mammalian-derived systems while providing improved stability and yield [87]. In addition, researchers have developed hyaluronic acid-coated extracellular vesicles from red cabbage, designated as t-Rabex, to serve as a targeted therapeutic approach for inflammatory bowel disease (IBD). This modification substantially improved the vesicles’ capacity to target and accumulate in gastrointestinal tissues relative to their unmodified versions. Importantly, t-Rabex exhibited a synergistic therapeutic effect by both inhibiting macrophage-mediated inflammation and facilitating the regeneration of colonic epithelial cells [88].

### 5.2. Drug Loading

PELNs can efficiently load small molecule drugs, including nucleic acids, proteins and chemotherapeutics through physical methods such as probe sonication, repeated freeze–thaw cycles or electroporation, or chemical approaches like hydrophobic insertion. These techniques could temporarily alter the permeability of vesicle membrane to achieve drug encapsulation without significantly compromising their native structure and targeting properties [89].

Zhang’s group found a natural siRNA delivery system utilizing edible ginger-derived lipid vehicles (GDLVs) to overcome the limitations associated with synthetic nanoparticles, such as toxicity and high cost, in the treatment of ulcerative colitis. When loaded with siRNA targeting CD98, these GDLVs exhibited colon-specific targeting capabilities and effectively reduced CD98 expression. Moreover, GDLVs were demonstrated to have favorable biocompatibility following oral administration in vivo [90]. Moreover, ginger-derived nanoparticles were also explored as a combination therapy for hereditary hemochromatosis comprising dietary iron restriction and oral administration of siRNA targeting the iron importer Dmt1. This intervention effectively diminished duodenal Dmt1 expression and markedly decreased serum and liver iron concentrations in adult hepcidin-deficient mice. Notably, the ginger-based delivery vehicle exhibited independent iron-lowering effects, indicating potential additional bioactive properties. The study established that this combined modality successfully reverses established iron overload in hereditary hemochromatosis murine model [91]. In addition, PELNs derived from broccoli were demonstrated to be used as an effective novel drug delivery system for therapeutic miRNAs. PELNs were successfully isolated from broccoli using a combination of ultracentrifugation and size-exclusion chromatography, and then loaded with exogenous miRNAs. The engineered PELNs showed efficient cellular uptake and induced toxicity in intestinal cells. The study indicated that PELNs from broccoli offered advantages such as gastrointestinal stability, cost-effectiveness, and low immunogenicity, making them promising candidates for future pharmaceutical applications involving RNA-based therapies [92].

**Figure 7:**
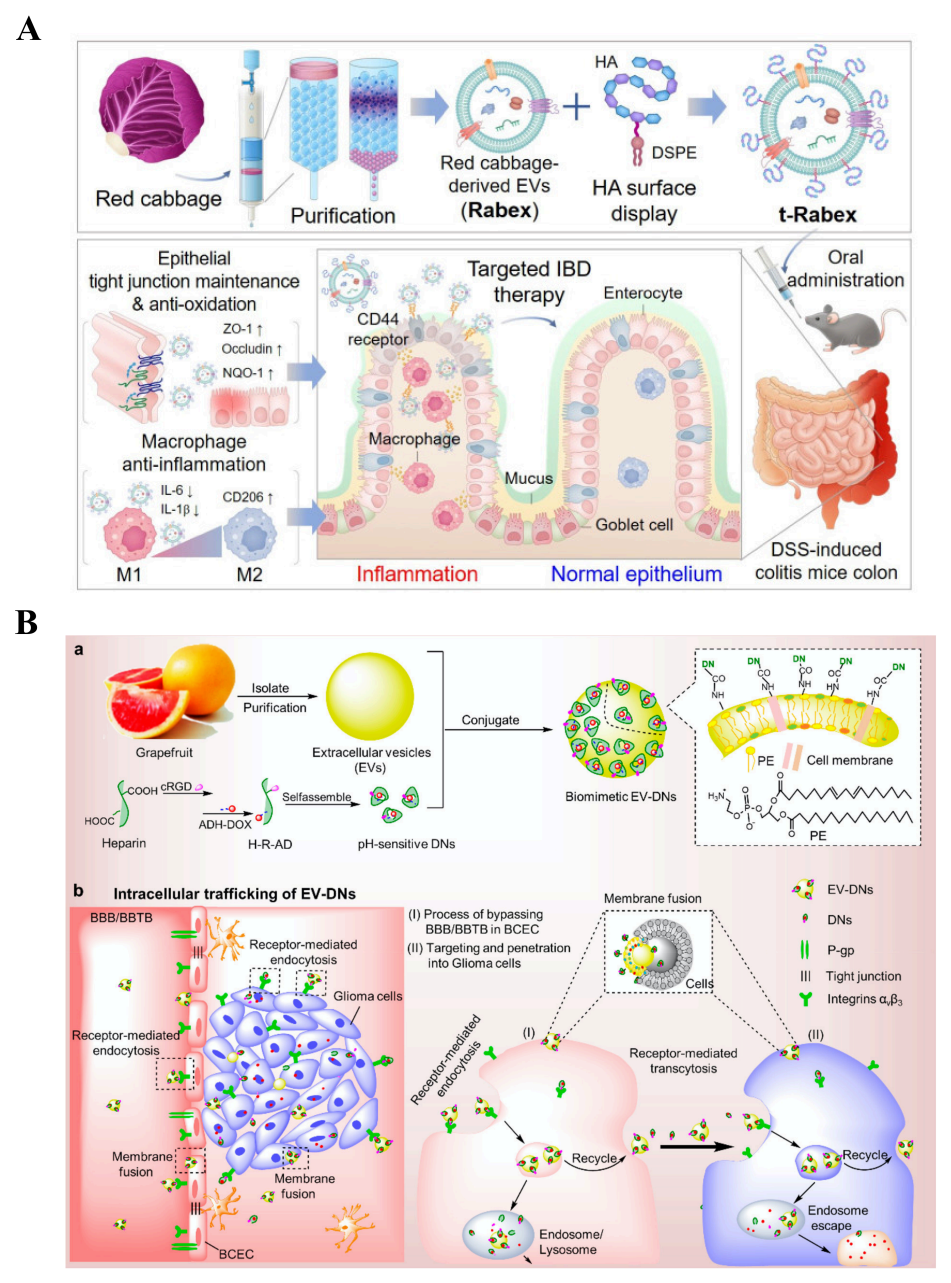
(**A**) Schematic illustration and mechanism of the surface engineered t-Rabex for the efficient and targeted IBD therapy. Obtained from Ref. [88]. Copyright © 2024, Su Jin Kang et al. (**B**) Schematic of the preparation of biomimetic delivery system of EV-DNs and the mechanism of EV-DNs bypassing blood–brain barrier/the blood–(brain tumor) barrier and penetrating glioma. Obtained from Ref. [93]. Copyright © 2021, Wenbo Niu et al.

Furthermore, a biomimetic delivery system combining grapefruit-derived extracellular vesicles (EVs) with pH-sensitive, heparin-based nanoparticles (DNs) loaded with doxorubicin (DOX) was developed to enhance blood–brain barrier/the blood–(brain tumor) barrier traversal and tissue penetration, in which heparin improves stability, and pH-sensitive DNs enable tumor-targeted drug release via cRGD ligands. By integrating EVs and DNs (EV-DNs), the system achieved efficient drug loading, targeted delivery, and enhanced therapeutic potential for glioma treatment in vivo [93] (Figure 7). Plant-derived exosomes (PEs) from Lycium barbarum L. were demonstrated to exhibit anti-inflammatory and neural differentiation-promoting properties, showing superior efficacy in enhancing neural differentiation. In spinal cord injury (SCI) treatment, a 3D-printed scaffold loaded with isoliquiritigenin (ISL)-encapsulated PEs (ISL@PE) effectively modulated post-injury inflammation and promoted axon regeneration, leading to improved neurological recovery in vivo [94]. Huang et al. successfully fabricated curcumin-loaded ginger-derived nanovesicles (CG) via PEG-assisted density gradient method with high efficiency. The orally administered CG specifically accumulated in the colon and exhibited superior therapeutic effects on ulcerative colitis mice compared to free curcumin or empty vesicles, which alleviated inflammation, improved disease markers, and positively regulated gut microbiota and metabolites [95].

### 5.3. Membrane Hybridization

Membrane fusion of PELNs with synthetic liposome or polymer can effectively improve the structural stability and loading capacity for therapeutic molecules of nanocarriers. This strategy is inspired by animal exosome engineering to overcome the inherent limitations of natural PELNs in terms of targeting specificity and therapeutic efficacy [96].

Engineered Panax ginseng-derived exosomes with neutrophil membranes loaded with miRNA 182-5p (N-exo-miRNA 182-5p) were developed as a novel therapeutic agent. The N-exo-miRNA 182-5p exhibited significant protective effects against sepsis-induced acute lung injury in both cellular and animal models through the targeted regulation of the NOX4/Drp-1/NLRP3 signaling pathway [97].

**Figure 8:**
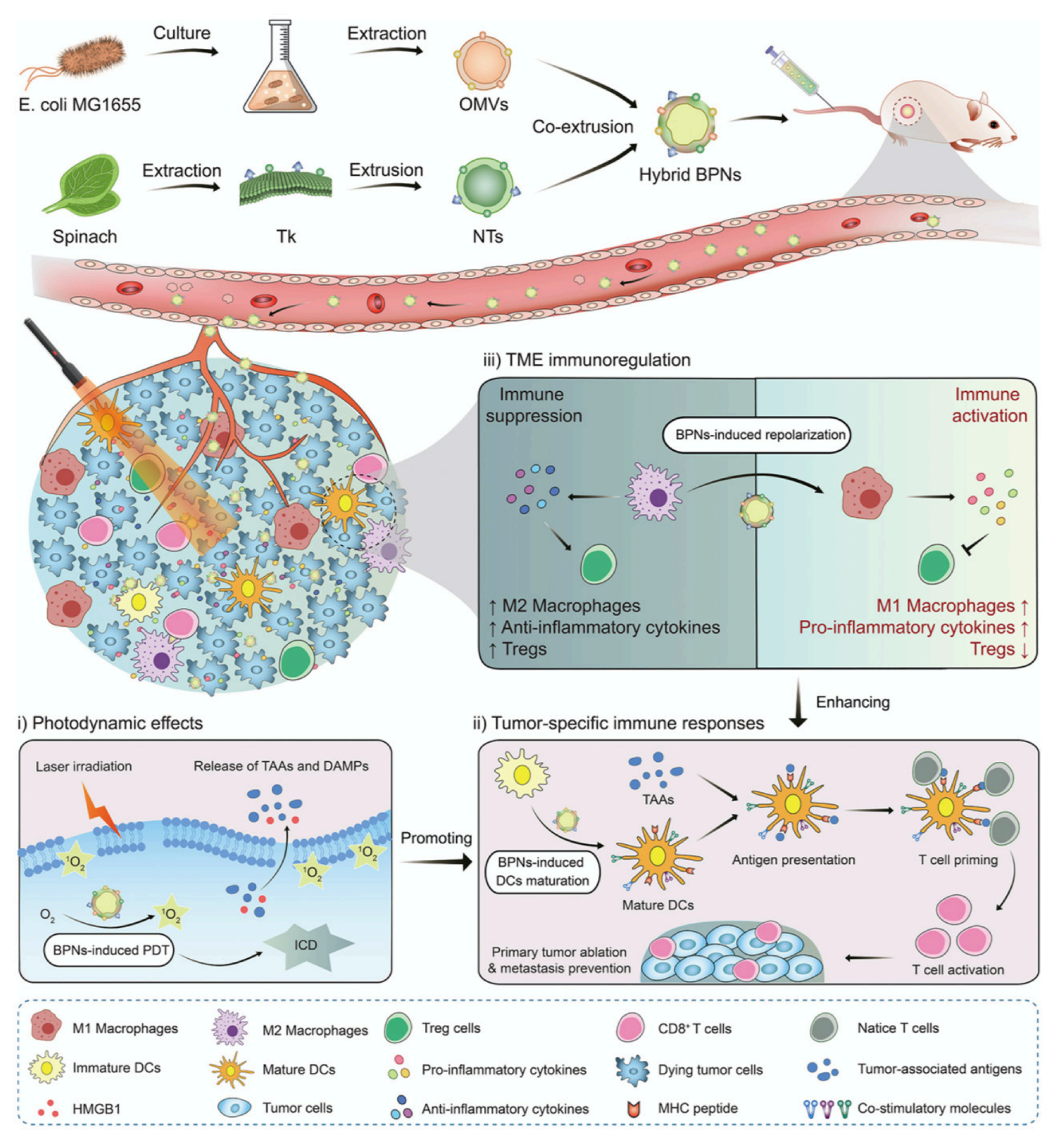
Schematic illustration of preparation and therapeutic strategy of BPNs. Obtained from Ref. [98]. Copyright © 2022, Wan-Ru Zhuang et al.

By integrating bacterial membrane vesicles with thylakoid-like nanovesicles derived from spinach chloroplasts, an in situ activatable vaccine system was constructed at tumor sites. The bacteria–plant hybrid nanovesicles (BPNs) showed enhanced tumor tissue-targeting precision, elevated immune cell activation levels, and improved tumor antigen presentation, thereby triggering a strong CD8^+^ T cell immune response. In addition, BPNs also exhibited regulatory effects on the tumor microenvironment and alleviated immunosuppression, comprehensively enhancing antitumor immunity in vivo [98] (Figure 8).

**Figure 9:**
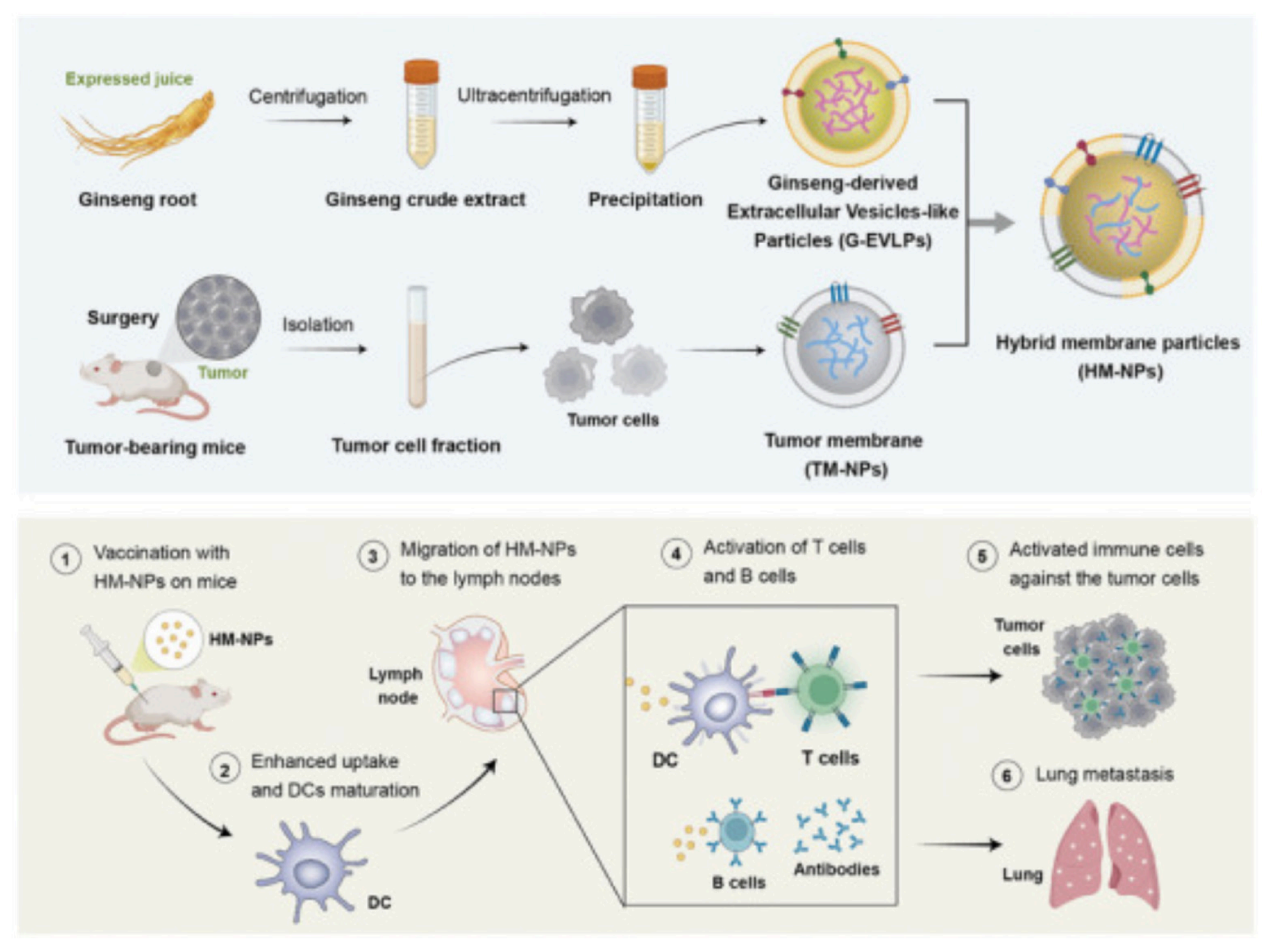
Schematic illustration of the construction and mechanistic process of the cancer vaccine HM-NPs. Obtained from Ref. [99]. Copyright © 2024, Haoran Wang et al.

Based on this research framework, we designed a functional cancer hybrid vesicle (HM-NPs) by fusing ginseng-derived extracellular vesicle-like particles (G-EVLPs) with patient-derived autologous tumor cell membranes. The hybrid vesicles effectively promoted dendritic cell uptake of tumor antigens and activated tumor-specific cytotoxic T lymphocytes, showing potential in preventing tumor recurrence and metastasis in vivo [99] (Figure 9).

### 5.4. Genetic Engineering

Transgenic technology allows for the targeted enhancement of specific functional molecules within PELNs, significantly improving their therapeutic potential. For example, genetic engineering approaches that induce overexpression of small RNAs with well-defined therapeutic targets in plants can enrich the resulting PELNs with these therapeutic nucleic acids. These engineered PELNs can be designed to more precisely modulate disease-related pathways, such as targeting hepatic insulin signaling pathways in metabolic disorders or regulating macrophage polarization in inflammatory diseases. This approach not only leverages the inherent biocompatibility and low immunogenicity of PELNs but also endows them with enhanced disease-targeting capabilities and therapeutic efficacy through customized molecular cargo, offering a promising strategy for developing next-generation smart plant-based nanomedicines [100]. For example, Ahrazem’s group demonstrated that crocin-rich extracts derived from genetically engineered “Tomafran” tomatoes, when encapsulated in chitosan nanoparticles or exosomes, exhibited significant neuroprotective effects. Even at low doses, these encapsulated forms effectively protected neuronal cells from damage in a model of neurodegeneration. The research suggested that this plant-based, exosome-mediated delivery system could offer an economical therapeutic approach with considerable potential for delaying or preventing neurodegenerative disorders such as Alzheimer’s disease [101].

## 6. Challenges in PELNs Application

Despite the promising prospects of plant exosomes, several challenges still remain. The manufacturing standardization of PELNs faces significant challenge. The extraction methods of PELNs are diverse, resulting in obviously batch-to-batch differences. Currently, the main obstacle is the lack of a unified production process that conforms to Good Manufacturing Practice (GMP). Therefore, it is necessary to develop efficient industrialized extraction technologies and ensure consistency and traceability of clinical trial materials by balancing yield, purity, and cost [102]. To achieve industrial-scale expansion of PELNs, the most suitable approach is a systematic process framework that integrates upstream controllable bioproduction with downstream efficient separation and purification. The core principle involves moving away from reliance on natural plant tissues to establish a standardized, scalable, and economically viable production system. Traditional methods of direct extraction from fruits or roots are constrained by seasonal variations, geographical origins, and batch-to-batch inconsistencies, rendering them unsuitable for industrialization. The most ideal alternative approach involves establishing a plant cell suspension culture system. Moreover, the yield of exosomes can be directionally enhanced or specific functions can be conferred through adjustment of cultivation conditions or genetic engineering approaches [103]. Studies have demonstrated that exosomes derived from different cultivation sources exhibit variations in both yield and compositional characteristics, thereby providing a basis for process optimization [104]. Subsequently, an integrated downstream processing approach combining tangential flow filtration with size-exclusion chromatography was employed to achieve efficient and scalable separation and purification. This technological pathway eliminates dependence on agricultural raw materials, enabling standardized, controlled, and scalable production processes, which represents the essential route for transforming laboratory-scale curiosities into stable, reliable commercial products [105].

The second pivotal challenge is the safety and toxicity of PELNs. Although most studies have reported that PELNs exhibited good biocompatibility in vitro and in animal models, safety assessment still requires caution. For example, tea-derived PELNs were observed to cause mild abnormalities in liver and kidney indicators in mouse models after intravenous administration. Some PELNs rich in specific lectins may activate the complement system or trigger immune responses [106]. Long-term safety data are particularly lacking, including their accumulation potential in vivo, long-term effects on the immune system, and possible reproductive toxicity. The International Organization for Standardization (ISO) has not yet established dedicated safety evaluation guidelines for PELNs, with current evaluations mostly referencing relevant standards for nanomedicines and biological products. A natural function of PELNs is the sequestration and transport of exogenous substances, including pesticides and fungicides that may be present in the environment. Therefore, when using fruits and vegetables from intensive agriculture as raw materials, the extracted PELNs could indeed co-concentrate these chemical residues. Without proper control measures, this process would transform what is originally a potentially health-beneficial product into a potentially harmful substance [107]. Research indicates that to ensure the safety and functionality of PDEVs products, measures must be implemented at both the source and process levels. Firstly, priority should be given to the use of raw materials from organic or controlled cultivation. Secondly, a rigorous quality control system during the isolation and purification of PELNs must be established, and stringent residue testing must be implemented [108]. Thirdly, to fundamentally address the issues of raw material quality fluctuations and contamination, controlled cultivation methods such as plant tissue culture techniques and temporary immersion bioreactor systems are considered highly promising solutions [109].

The third challenge of PELNs is the clinical translation. Although PELNs have shown great cross-kingdom therapeutic potential, their clinical translation is at a critical juncture. Currently, only a few trials are underway (e.g., NCT04879810 for evaluating curcumin or ginger-derived exosomes in IBD treatment), but these trials are still ongoing and no large population data are available yet [110]. At the same time, the lack of clarity in regulatory frameworks hinders practical implementation as standardized criteria have not been established regarding pharmaceutical classification nomenclature, quality control parameters, and safety thresholds for routes of administration, which poses a major obstacle to industrial-scale production [111]. The translational potential of PELNs is constrained by challenges in reproducibility of production processes, product uniformity and stability, as well as the absence of quality control systems compliant with pharmaceutical standards. Firstly, batch reproducibility represents one of the core bottlenecks hindering the transition of plant-derived extracellular vesicles from laboratory research to clinical applications. To ensure batch consistency, prioritize the use of fresh, minimally processed plant materials as raw ingredients, since traditional Chinese medicine processing methods may cause structural damage to exosome membranes and degradation of bioactive molecules. Secondly, Accurate identification is the prerequisite for ensuring product consistency and safety. The combination of transmission electron microscopy (TEM), nanoparticle tracking analysis (NTA), and protein immunoblotting (Western blotting) was employed to observe morphology, determine particle size and concentration, as well as identify characteristic molecular markers [112]. Thirdly, as bioactive nanoparticles, the long-term stability of plant-derived exosomes is critical for product commercialization. Storage must not only maintain the physical integrity of particles but also ensure the retention of biological activity in their payload. Currently, there is a lack of established and widely accepted methods for assessing their “efficacy” through biological activity assays [113]. Finally, efficacy determination is directly related to the therapeutic effects of plant exosomes. For specific therapeutic applications, establish corresponding bioactivity benchmarks and indices of therapeutic potential. In addition, functional indicator monitoring including monitor cell uptake efficiency, retention rate of active molecules, and in vivo circulation half-life of exosomes need to be explored [114]. Therefore, to serve as pharmaceuticals or medical devices, a rigorous quality control system must be established. Standard operating procedures (SOPs) spanning from raw material cultivation and harvesting to isolation and purification processes should also be established, ultimately resulting in production under GMP conditions. Functional homogenization and enhanced targeting can be achieved through surface modification or cargo loading of plant-derived extracellular vesicles, while maintaining rigorous quality control throughout the engineering process itself. Collaborative efforts among academia, industry, and regulatory authorities are required to develop appropriate quality standards, non-clinical evaluation frameworks, and clinical development guidelines for these novel biological therapeutic products derived from PELNs. Addressing these challenges is the only viable path to transform PELNs from promising “natural nanomaterial” into safe, effective, and reliable therapeutic product.

The mechanistic understanding of PELNs in vivo presents great challenge. The cross-kingdom communication mechanisms of PELNs remain largely elusive. Although it is established that they can enter mammalian cells through endocytosis, membrane fusion, and other pathways to deliver bioactive components such as proteins, lipids, and RNA, the specific molecular targets and signaling pathway networks involved remain unclear. The pharmacokinetic behavior of PELNs in vivo has also been insufficiently investigated, including systematic data regarding their biodistribution patterns, metabolic clearance pathways, and half-lives across different administration routes. This knowledge gap hinders rational design of dosage regimens and determination of optimal administration intervals. Integrating transcriptomics, proteomics, metabolomics, and bioinformatics analyses is required to establish a systems biology model for elucidating PELNs’ mechanisms of action [115]. PELNs as drug delivery carriers can significantly enhance their targeting specificity, drug loading capacity, and circulation time through surface modification and drug loading strategies. However, these engineering modifications also introduce a series of limitations, which are primarily manifested in the following aspects. Firstly, excessive surface modification may alter membrane fluidity, rendering exosomes more susceptible to degradation during storage or in vivo circulation [116]. Secondly, modified exosomes may exhibit increased sensitivity to temperature, pH variations, or enzymatic degradation, which could compromise their long-term storage stability and in vivo functionality [117]. Thirdly, certain chemical coupling agents or drugs loaded at high concentrations may be locally released following exosome degradation, potentially inducing cytotoxicity or systemic toxicity [118]. Finally, the metabolic pathways, accumulation potential, and long-term immunological effects of engineered plant exosomes in vivo remain insufficiently investigated, particularly with regard to the risks associated with cross-species applications requiring further evaluation [119].

Furthermore, optimizing the storage stability of PELNs still faces challenges. The PELNs exhibit extreme temperature sensitivity due to their phospholipid bilayer structure, necessitating frozen storage at −80 °C or −20 °C. Failure to maintain these temperatures results in structural disintegration and precipitous concentration decline within hours under ambient conditions (approximately 22 °C), with shelf life limited to mere weeks. To overcome the constraints of high-cost cold chain logistics and bioactive degradation, researchers are actively exploring strategies including incorporation of protective agents, or application of freeze-drying technology, aiming to extend formulation stability for room-temperature preservation and thereby facilitate large-scale commercialization and clinical translation [120].

## 7. Future Outlook

For the future directions of PELNs, further research will move beyond traditional extraction methods, focusing on developing efficient, scalable, and GMP-compliant production processes to ensure product uniformity and reproducibility. More importantly, engineering modifications will endow PELNs with “smart” functionalities. For example, genetic engineering can be used to modify donor plants for natural production of PELNs carrying specific therapeutic RNAs. Alternatively, surface modification is also expected to enhance their active targeting capability toward diseased tissues, thereby improving the therapeutic efficacy while reducing off-target toxicity [121].

In addition, there is an urgent need to move from proof-of-concept pre-clinical studies towards well-designed clinical trials. Future research should prioritize areas with clear pathological mechanisms and unmet clinical needs for breakthroughs, such as using the inherent anti-inflammatory properties and intestinal barrier repair capabilities of PELNs to conduct efficacy and safety evaluations of oral formulations for IBD treatment [122]. In addition, engineered PELNs loaded with chemotherapeutic drugs or immunomodulatory molecules can be combined with existing therapies to evaluate their synergistic effects in overcoming drug resistance and modulating the tumor microenvironment [123].

PELNs, as an emerging drug delivery vehicle, demonstrate significant advantages in bioavailability, particularly within the field of oral administration. Their key advantages including excellent gastrointestinal stability, immune tolerance, targeted delivery potential and good biocompatibility [124]. In addition, PELNs could interact with target cells through both endocytic pathways and direct membrane fusion. The specific internalization routes are influenced by multiple factors, including the size and surface charge of PELNs, their lipid and protein composition, as well as the type and physiological state of the target cells [125]. As natural nanocarriers, the application of PELNs should not be confined to small-molecule drug delivery. Future research should actively explore their potential in emerging fields, such as serving as gene therapy vectors for delivering CRISPR-Cas9 components or functioning as vaccine delivery systems to present antigens and activate mucosal immunity, thereby offering novel strategies for infectious disease prevention and tumor immune-prophylaxis [126,127,128].

In summary, PELNs possess an inherent nanoscale structure and excellent biocompatibility, enabling them to effectively encapsulate and deliver various therapeutic molecules, including small-molecule chemical drugs, nucleic acids, and proteins. Compared to synthetic nanocarriers, PELNs typically exhibit lower toxicity, reduced immunogenicity, enhanced cellular uptake efficiency, and their targeting specificity can be further improved through surface functionalization. Moreover, PELNs possess multiple biological activities and can serve as “natural medicines” or adjuvant therapeutic agents, including tissue repair, antitumor, anti-inflammatory and antioxidant activities. Furthermore, PELNs offer a novel translational direction for disease treatment, particularly in the context of precision medicine and personalized therapy. Current studies have investigated the therapeutic effects of PELNs in models of cancer, neurodegenerative diseases, liver injury, and intestinal inflammation, thereby laying the foundation for their progression into clinical trials.

## 8. Conclusions

PELNs, as an emerging biomedical tool which integrates the advantages of natural origins with the precision of nanotechnology, show great promise in drug delivery, disease treatment, and prevention. Their unique biochemical composition, low immunogenicity, and cross-species regulatory capabilities make them a promising platform for precision medicine. However, overcoming production, safety, and translational challenges are the key challenges. With multidisciplinary collaboration and continued research, PELNs are poised to revolutionize therapeutic strategies, offering green and effective solutions for a wide range of diseases.

## Figures and Tables

**Table 1:** Comparison of the separation techniques.

Extraction Method	Impact on Vesicle Quality	Impact on Biological Interpretation	Advantages	Disadvantages	Ref.
Ultracentrifugation	Repeated centrifugation damages integrity and bioactivity; impurities (e.g., fibers, starch) cause insufficient purity.	Compromised activity or co-isolated impurities may mask or mislead interpretation of therapeutic potential.	Simple procedure; low contamination risk; gold standard; successfully used for various plants and disease models.	Repeated centrifugation damages integrity/activity; harsh conditions and high cost; low purity due to impurities; purity increase reduces yield.	[25,26,27]
Density gradient centrifugation	High purity, good structural integrity, effective preservation of bioactivity.	Suitable for precise quantification and mechanistic studies requiring high purity; low yield may limit some downstream applications.	High purity; intact structure; suitable for high-purity research.	Low yield and time-consuming; cannot fully remove macromolecular contaminants; limited scalability.	[28,29,30]
Ultrafiltration	Membrane clogging or vesicle entrapment reduces recovery; inability to remove small impurities.	Unstable recovery may affect reproducibility; residual impurities may interfere with assays.	Fast and easy; no special equipment; size selection possible; handles large volumes; maintains vesicle activity.	Membrane clogging and vesicle trapping; cannot remove tiny impurities; requires sample pretreatment and careful centrifugal control.	[31,32]
Size-exclusion chromatography	High purity, intact structure, good bioactivity retention; sample dilution reduces recovery.	High purity facilitates analysis of authentic composition and function; low recovery challenges in vivo studies.	High purity, intact structure, good activity; suitable for downstream analysis; combinable with other methods; scalable; commercial columns are fast and reusable.	Traditional SEC: complex, time consuming, sample dilution; cannot completely remove impurities of similar size.	[33,34,35]
Polymer precipitation	Gentle process with minimal vesicle damage; residual polymers and co-precipitated proteins cause low purity.	Residual polymers and impurities may interfere with cellular uptake, signaling pathways, and cause false-positive results.	Simple, low-cost, fast; minimal vesicle damage; scalable.	Low purity (co-precipitates proteins, etc.); needs combination with other methods to improve purity.	[36,37]
Microfluidics	Fast processing, low sample consumption; current throughput is limited, consistency and standardization insufficient.	Suitable for high-throughput screening or small-sample analyses; insufficient stability may affect comparability of functional studies.	Miniaturized, automated, high-throughput; low sample consumption; fast processing; integrates separation, recovery and detection.	Low throughput and limited capacity; complex and lacks standardization; early stage; needs better compatibility and stability.	[26,38,39]

**Table 2:** Summary of applications of PELNs in biotherapeutics.

Biological Activity	Disease	Plant Source	Main Findings	Ref.
Antitumor	Triple-negative breast cancer	Ginger	Induce apoptosis, generate reactive oxygen species (ROS), and arrest the cell cycle.	[51]
	Chronic myeloid leukemia	*Citrus limon*-L	Activate the TRAIL-mediated apoptosis pathway, thereby inhibiting cancer cell proliferation.	[52]
	Colorectal cancer		Activate the macropinocytosis pathway to inhibit colorectal cancer cell growth, remaining effective even in p53-inactivated cancer cells.	[53]
	Non-small cell lung cancer	Cucumber	Inhibit STAT3 activation, induce ROS production, block cell cycle progression, and activate caspases.	[54]
	Melanoma	Ginseng	Shifting macrophage polarization towards the M1 phenotype triggers apoptosis in melanoma cells via ROS.	[55]
	Glioma		Promoting M1 macrophage infiltration and reprogramming the immune microenvironment to suppress tumor growth.	[56]
	Colorectal cancer		Reprogrammed TAMs promote the secretion of CCL5/CXCL9, which synergizes with PD-1 mAb to recruit CD8+ T cells into the tumor.	[57]
	Oral squamous cell carcinoma	Bitter melon	The reversal of 5-FU resistance in oral squamous cell carcinoma is achieved through the induction of reactive oxygen species (ROS) and inhibition of NOD-like receptor protein 3 (NLRP3), thereby generating a synergistic anticancer effect.	[58]
Anti-inflammatory	Colitis	Ginger	Targeting the gut-specific pathway and inhibiting miRNA to suppress NF-κB signaling attenuates LPS-induced intestinal inflammation.	[60]
		Tea leaves	Galactosylated nanoparticles, designed to target macrophages, impede the progression of IBD-associated carcinogenesis by mitigating inflammation and restoring barrier function.	[61]
	Ulcerative colitis	*Momordica charantia*	Inhibit the inflammatory response in macrophages, scavenge ROS, and protect cells from oxidative damage.	[62]
		Garlic	peu-MIR2916-p3 specifically promotes the growth of Bacteroides thetaiotaomicron, remodels the gut microbiota and attenuates colitis.	[63]
	Neuro-inflammatory	Allium	Upregulation of HO-1 and downregulation of iNOS transcription inhibits microglial inflammation, with dexamethasone loading exerting a synergistic effect.	[64]
		Atractylodes lancea	Effective attenuation of LPS-induced microglial neuroinflammation via multi-target modulation of pro-inflammatory mediators (NO, IL-1β, IL-6, TNF-α).	[65]
Regenerative effect	Anti-hair loss	*Iris germanica* L	The activation of the Wnt/β-catenin pathway, mediated via GSK-3β/AKT/ERK signaling, mitigates oxidative stress and mitochondrial damage, thereby preventing and treating hair loss.	[66]
	Skin regeneration	Apple	Inhibition of the TLR4/NF-κB inflammatory pathway and promotion of collagen synthesis.	[67]
	Skin photoaging	Olive leaf	Inhibition of the miR168a-5p-mediated NF-κB inflammatory senescence signaling pathway mitigates skin injury and enhances repair.	[68]
		Aloe	Activation of the Nrf2/ARE pathway alleviates UV-induced oxidative stress and DNA damage, thereby delaying skin photoaging.	[69]
	Cutaneous repair	Ginseng	Promote skin cell proliferation, migration, and angiogenesis through the ERK and AKT/mTOR pathways, while increasing secretion of key extracellular matrix components.	[70]
	Wound healing	Coriander	Activation of the Nrf2 pathway enhances antioxidant and anti-inflammatory effects.	[71]
		Wheat	Promote proliferation and migration of endothelial/epithelial/fibroblast cells, inhibits apoptosis, and enhances collagen deposition and angiogenesis.	[72]
		Morinda Officinalis	Activation of the MAPK/YAP1 pathway enhances endothelial function, and hydrogel-mediated delivery accelerates wound healing.	[73]
Metabolism regulation	Obesity	Garlic	Inhibition of the c-Myc/STING pathway attenuates neuroinflammation and promotes neuronal differentiation and survival.	[75]
	Type 2 diabetes	Garlic	Regulation of GLP-1 and Inhibition of the cGAS/STING Pathway.	[76]
		Ginger	Restoration of Foxa2 signaling homeostasis in the intestinal epithelium prevents high-fat diet-induced insulin resistance.	[77]
		Tangerine-peel	Modulation of the gut microbiota and enhancement of hepatic lipid metabolism.	[78]
	Nonalcoholic fatty liver disease	Blueberry	Activation of the Nrf2 pathway enhances antioxidant defense, suppresses hepatic lipogenic genes, thereby improving mitochondrial function and attenuating lipid accumulation.	[79]
Neurodegenerative disease	Ferroptosis	Green onion	Regulation of iron metabolism protein expression, upregulation of GPX4, and enhancement of glutathione synthesis.	[80]
	Alzheimer’s disease	*Citrus limon*	Protect neuronal cells from β-amyloid protein-induced oxidative damage through its antioxidant activity.	[81]
		Lycium ruthenicum Murray	Inhibition of the MAPK and PI3K/AKT signaling pathways mitigates Aβ-induced apoptosis in PC12 cells.	[82]
	Parkinson’s disease	Gardenia	Inhibition of the p38 MAPK/p53 pathway reduces mitochondrion-dependent apoptosis in dopaminergic neurons.	[83]
	Ischemic Brain Injury	*Momordica charantia*	Inhibition of matrix metalloproteinase-9 (MMP-9) and activation of the AKT/GSK3β signaling pathway exert neuroprotective effects.	[84]

## Data Availability

No data was used for the research described in the article.
